# Estimating the burden of multiple endemic diseases and health conditions using Bayes’ Theorem: A conditional probability model applied to UK dairy cattle

**DOI:** 10.1016/j.prevetmed.2022.105617

**Published:** 2022-06

**Authors:** Philip Rasmussen, Alexandra P.M. Shaw, Violeta Muñoz, Mieghan Bruce, Paul R. Torgerson

**Affiliations:** a Vetsuisse Faculty, University of Zurich, Zurich, Switzerland; b Department of Livestock and One Health, University of Liverpool, Liverpool, United Kingdom; c School of Veterinary Medicine, Centre for Biosecurity and One and Health, Murdoch University, Murdoch, Australia

**Keywords:** GBADs, AHLE, Economic, Impact, Endemic, Disease, Livestock, Bayes’ Theorem, Comorbidity, Dairy

## Abstract

The Global Burden of Animal Diseases (GBADs) is an international collaboration aiming, in part, to measure and improve societal outcomes from livestock. One GBADs objective is to estimate the economic impact of endemic diseases in livestock. However, if individual disease impact estimates are linearly aggregated without consideration for associations among diseases, there is the potential to double count impacts, overestimating the total burden. Accordingly, the authors propose a method to adjust an array of individual disease impact estimates so that they may be aggregated without overlap. Using Bayes’ Theorem, conditional probabilities were derived from inter-disease odds ratios in the literature. These conditional probabilities were used to calculate the excess probability of disease among animals with associated conditions, or the probability of disease overlap given the odds of coinfection, which were then used to adjust disease impact estimates so that they may be aggregated. The aggregate impacts, or the yield, fertility, and mortality gaps due to disease, were then attributed and valued, generating disease-specific losses. The approach was illustrated using an example dairy cattle system with input values and supporting parameters from the UK, with 13 diseases and health conditions endemic to UK dairy cattle: cystic ovary, disease caused by gastrointestinal nematodes, displaced abomasum, dystocia, fasciolosis, lameness, mastitis, metritis, milk fever, neosporosis, paratuberculosis, retained placenta, and subclinical ketosis. The diseases and conditions modelled resulted in total adjusted losses of £ 404/cow/year, equivalent to herd-level losses of £ 60,000/year. Unadjusted aggregation methods suggested losses 14–61% greater. Although lameness was identified as the costliest condition (28% of total losses), variations in the prevalence of fasciolosis, neosporosis, and paratuberculosis (only a combined 22% of total losses) were nearly as impactful individually as variations in the prevalence of lameness. The results suggest that from a disease control policy perspective, the costliness of a disease may not always be the best indicator of the investment its control warrants; the costliness rankings varied across approaches and total losses were found to be surprisingly sensitive to variations in the prevalence of relatively uncostly diseases. This approach allows for disease impact estimates to be aggregated without double counting. It can be applied to any livestock system in any region with any set of endemic diseases, and can be updated as new prevalence, impact, and disease association data become available. This approach also provides researchers and policymakers an alternative tool to rank prevention priorities.

## Highlights

• Three approaches to impact aggregation for multiple endemic diseases explored.
• Flexible method proposed to avoid double counting impacts within a livestock system.
• Illustrated using 13 endemic diseases and health conditions in UK dairy cattle.
• Provides an alternative perspective on ranking disease prevention priorities.

## 1 Introduction

The Global Burden of Animal Diseases (GBADs) is a multi-institutional collaborative programme aiming to provide information for evidence-based investment plans, facilitate the allocation of resources to key social, economic, and environmental problems, and support high quality evaluation of existing animal health investments (GBADs, 2021). The formulation of effective animal health policy requires a consistent and comparable description of animal diseases, the risk factors associated with them, and the effectiveness of potential intervention practices (Rushton et al., 2018). Accordingly, one objective of GBADs is to estimate the economic burden associated with endemic diseases, both communicable and non-communicable, in livestock production systems. From the human health perspective, analogous efforts to estimate and attribute disease burdens have been undertaken by other research programmes. For example, the Global Burden of Disease (GBD) project through its partnership with the Institute for Health Metrics and Evaluation (IHME, 2020), the Foodborne Disease Burden Epidemiology Reference Group (FERG, 2021), and the European Burden of Disease Network (COST, 2021).

There are several possible approaches to estimating the total economic burden attributable to a set of diseases and health conditions. A straightforward approach is the direct linear aggregation of disease- and condition-specific economic losses (e.g., annual monetary losses per animal), where estimates from epidemiologic and economic studies are combined with prevalence estimates to directly estimate the losses attributable to each disease within a region or livestock system. These disease-specific losses are then summed to obtain an estimate of the total economic burden. However, because these economic loss estimates often overlook the impacts of other diseases existing within the study population, when multiple disease-specific estimates are linearly aggregated, there is the potential to double count impacted animals and therefore overestimate economic losses. This potential for overestimation due to double counting is discussed from the human health perspective in Honeycutt et al. (2011) and from the animal health perspective in Torgerson and Shaw (2021), which echoes the concerns with a direct approach to aggregation that will be discussed herein. While the GBD, FERG, and COST approaches focus on disability-adjusted life years (DALYs), as proposed by Murray (1994), which quantify the burden of human disease based on the number of life years potentially lived in optimal health (Devleesschauwer et al., 2014), this measure does not translate to a livestock framework without some adaptation. For example, Torgerson et al. (2018) propose a modified measure called a zDALY that quantifies animal production losses due to zoonotic diseases through a time trade-off for human life-years, while Torgerson and Shaw (2021) suggest that the maximum potential livestock productivity in a “utopian” scenario and the current observed productivity form the upper and lower bounds, respectively, of an animal health loss envelope (AHLE). This AHLE, or the gap attributable to poor animal health, contains, by definition, the entire burden due to animal health problems, including not only losses due to endemic diseases and health conditions, but also losses due to health issues such as malnutrition, some injuries, and predation (Huntington et al., 2021). By considering this hypothetical upper limit to productivity, the potential for overestimation of this entire AHLE is reduced (Rushton et al., 2021).

Continuing within the AHLE theoretical framework, an alternative approach to direct linear aggregation is to use the observed mean values of key production characteristics (e.g., yield in terms of kg of output, fertility in terms of birthing interval days, mortality in terms of culling rate, etc.) and estimates of the impacts of the diseases and conditions on those production characteristics (e.g., reduction in yield, increase in birthing interval, increase in culling rate, etc.) to solve for the disease-free or “healthy” value of that production characteristic. The difference between the disease-free value and the observed mean value would be the productivity gap attributable to the diseases and conditions being modelled, and these gaps can be valued and attributed according to the relative impacts of those diseases and conditions. This approach is less prone to misestimation as it is anchored by the observed mean and relies on productivity impact estimates that are less likely to be affected by regional economic differences (e.g., currency values, farm-gate prices, wage rates, interest rates, replacement prices, salvage prices, etc.) than economic loss estimates.

While this productivity gap attribution approach is a viable alternative to direct linear aggregation, it still does not capture the impacts of associations between diseases within the production system and is thus still prone to double counting. The authors propose a flexible and convenient method to adjust an array of individual disease impact estimates so that they may be aggregated without overlap. Using Bayes’ Theorem, conditional probabilities are derived from inter-disease odds ratios (ORs) in the literature, which capture the statistical associations between diseases or health conditions. ORs are frequently presented in medical reports and literature as estimates of the direction and magnitude of the relationship between binary variables (e.g., exposure and outcome), and they enable researchers to analyse the effects of other variables on that relationship using logistic regression (Bland and Altman, 2000, Martinez et al., 2017). These conditional probabilities derived from ORs are used to calculate the “excess probability” of disease occurrence across groups of animals with and without statistically associated diseases. While these excess probabilities are comparable to the risk difference (RD) or attributable risk (AR) across groups, they measure different things and are calculated in different ways. RDs or ARs measure the proportion of disease occurrence that can be attributed to a certain exposure (Hoffman, 2019) and are calculated by comparing risk across exposed and unexposed groups without consideration for ORs. On the other hand, the excess probabilities used in the proposed method measure the difference in the probability of disease occurrence across groups of animals and are calculated directly from ORs. Whereas RDs or ARs are interpreted as the excess risk of disease that can be attributed to a risk factor, these excess probabilities are interpreted as the probability of disease overlap given the odds of coinfection and can therefore be used to adjust disease impact estimates from the literature for these overlaps and the conflation of disease impacts that occurs as a result.

In this study, the authors propose a method to de-conflate impact estimates and use these de-conflated impacts to estimate the productivity, fertility, and mortality gaps attributable to disease. These gaps are then valued and attributed to the diseases and conditions being modelled, and once aggregated, estimate the total economic burden of an array of diseases and conditions within a livestock system. The authors also use Monte Carlo analyses to estimate the sensitivity of total losses to variations in the prevalence of the diseases and conditions, providing an alternative approach to ranking prevention priorities while also identifying potentially impactful inter-disease associations. Lastly, the complete methodology is illustrated using an example dairy cattle system with input values and supporting parameters from the UK, with 13 diseases and health conditions endemic to UK dairy cattle: cystic ovary, disease caused by gastrointestinal nematodes (GIN), displaced abomasum, dystocia, fasciolosis, lameness, mastitis, metritis, milk fever, neosporosis, paratuberculosis, retained placenta, and subclinical ketosis.

## 2 Materials and methods

The proposed method, and an example of its application, is comprised of six distinct parts: 1) The logical foundation of the proposed method; 2) the de-conflation of disease impact estimates; 3) use of these de-conflated impact estimates to estimate the productivity, fertility, and mortality gaps attributable to disease; 4) application of the model to an example dairy cattle system; 5) comparison to other aggregation methods; and 6) sensitivity analyses.

### 2.1 Logical Foundation

Consider the following inter-disease OR relating diseases i and k:

(1) $$OR_{ik}=\frac{P(i\left|k\right)}{1-P(i\left|k\right)}/\frac{P\left(i\right|\neg k)}{1-P\left(i\right|\neg k)}$$

where, P(i|k) is the conditional probability of i given k and P(i|¬k) is the conditional probability of i given not k. This equation captures the statistical association between diseases i and k through the ratio of the odds of disease i given the presence of disease k to the odds of disease i given the absence of disease k. Now consider the following inequality, which represents a situation where there is a positive statistical association between diseases i and k:

(2) $${\mathrm{OR}}_{\mathrm{ik}}>1$$

Eq. (1) and inequality (2) can be used to compare P(i|k) and P(i|¬k):

(3) P(i|k)1−P(i|k)/P(i|¬k)1−P(i|¬k)>1⇒P(i|k)1−P(i|k)>P(i|¬k)1−P(i|¬k)⇒P(i|k)*[1−P(i|¬k)]>P(i|¬k)*[1−P(i|k)]⇒P(i|k)−P(i|k)*P(i|¬k)>P(i|¬k)−P(i|k)*P(i|¬k)⇒P(i|k)>P(i|¬k)

Therefore, from (1) through (3), given a positive statistical association between diseases i and k, the probability of disease i in a sample of animals with disease k is greater than the probability of disease i in a sample of animals without disease k. In other words, given an inter-disease ${\mathrm{OR}}_{\mathrm{ik}}$ > 1, there is an excess probability of disease i among animals with disease k. The proposed method considers a livestock system at a representative, average point in time. This assumption that we are taking a “snapshot” of a livestock system implies that the entire period being modelled is compressed into that point in time, and that causal associations can also be interpreted as purely statistical associations and therefore also result in disease overlap. In other words, if disease i is a predisposing condition for disease k with a causal relationship that manifests itself within the period being captured by the snapshot, then at that representative point in time, an animal will have both i and k, and vice versa.

Suppose that we also have independently generated impact estimates for both diseases (e.g., reduced output, reduced fertility, increased mortality, etc.) from the literature. If we were to simply aggregate the product of each disease’s probability and their respective impacts and treat this aggregation as the total impact of the pair of diseases, we would be ignoring these excess probabilities of disease inherent to the samples that generated the impact estimates. If inter-disease ORs are greater (or less) than 1, then impact estimates can be conflated by the impacts of other diseases that are more (or less) prevalent in the case sample. Therefore, before the impacts of multiple diseases can be aggregated, disease impact estimates must be de-conflated to account for these disease probability differences.

### 2.2 De-conflation

Since inter-disease odds ratios pose a potential problem for aggregation, disease impact estimates must first be de-conflated. Continuing with disease pair i and k, there are only two possible combinations that sum to the probability of i: i|k and i|¬k. In other words, P(i) must equal the weighted sum of P(i|k) and P(i|¬k). Therefore, P(i|¬k) can be rewritten in terms of P(i|k):

(4) P(i)=P(i|k)*P(k)+P(i|¬k)*[1−P(k)]⇒P(i|¬k)*[1−P(k)]=P(i)−P(i|k)*P(k)⇒P(i|¬k)=P(i)−P(i|k)*P(k)1−P(k)

Let γ=P(i|k). ${\mathrm{OR}}_{\mathrm{ik}}$ can also be rewritten:

(5) ORik=γ1−γ/P(i)−γ*P(k)1−P(i)−P(k)+γ*P(k)=γ*[1−[P(i)+P(k)]+γ*P(k)](1−γ)*[P(i)−γ*P(k)]=γ−γ*[P(i)+P(k)]+γ2*P(k)P(i)−γ*[P(i)+P(k)]+γ2*P(k)

Eq. (5) implies the following:

(6) $${\mathrm{OR}}_{\mathrm{ik}}*\left[P\left(i\right)-\gamma *\left[P\left(i\right)+P\left(k\right)\right]{+\gamma }^{2}*P\left(k\right)\right]=\gamma -\gamma *\left[P\left(i\right)+P\left(k\right)\right]{+\gamma }^{2}*P\left(k\right)$$

Which implies the following:

(7) $$\left({\mathrm{OR}}_{\mathrm{ik}}-1\right)*P\left(k\right)*\gamma^{2}-\left[\left({\mathrm{OR}}_{\mathrm{ik}}-1\right)*\left[P\left(i\right)+P\left(k\right)\right]+1\right]*\gamma +{\mathrm{OR}}_{\mathrm{ik}}*P\left(i\right)=0$$

Eq. (7) describes a quadratic function of γ where:

(8) $$a=\left({\mathrm{OR}}_{\mathrm{ik}}-1\right)*P\left(k\right)$$

(9) $$b=-\left[\left({\mathrm{OR}}_{\mathrm{ik}}-1\right)*\left[P\left(i\right)+P\left(k\right)\right]+1\right]$$

(10) $$c={\mathrm{OR}}_{\mathrm{ik}}*P\left(i\right)$$

Therefore, the value of γ can be calculated by solving for the roots of the quadratic function:

(11) $$\gamma =\frac{-b\pm \sqrt{b^{2}-4*a*c}}{2*a}$$

where only a single root generates a plausible conditional probability between 0 and 1. Given P(i|k)=γ, Bayes’ Theorem, which states that $P(i|k)=\frac{P(\mathrm{k|i})*P(i)}{P(k)}$, is used to solve for the additional conditional probabilities required to estimate the excess probabilities of disease across associated disease pairs:

(12) $$P(k|i)=\gamma *\frac{P\left(k\right)}{P\left(i\right)}$$

(13) $$P(k|\neg i)=\frac{P\left(k\right)*(1-\gamma )}{1-P\left(i\right)}$$

The excess probability of disease k among animals with disease i, or ${\mathrm{ep}}_{\mathrm{ki}}$, is estimated using the following equation:

(14) $$ep_{ki}=P(k|i)-P(k|\neg i)$$

If both diseases i and k impact the same production characteristic (e.g., some measure of output, fertility, mortality, etc.), then it is assumed that the raw impact estimate for disease i from the literature $m_{i}$ is conflated by the raw impact estimate of disease k from the literature $m_{k}$ due to the excess probability of disease k among animals with disease i and vice versa from Eq. (14). Assuming that the de-conflated impacts are in the same proportion as the raw impacts from the literature, the impact estimate for disease i is approximately de-conflated from the impact of disease k using the following equation:

(15) $$m_{\mathrm{ik}}=\frac{m_{i}}{1+\frac{{\mathrm{ep}}_{\mathrm{ki}}*m_{k}}{m_{i}}}$$

where $m_{\mathrm{ik}}$ equals the impact of disease i de-conflated from the impact of disease k. This process is then expanded to de-conflate i from the impact of all diseases j that it is associated with:

(16) $$m_{\mathrm{ij}}=\frac{m_{i}}{1+\frac{\sum_{j=1}^{n}\left({\mathrm{ep}}_{\mathrm{ji}}*m_{j}\right)}{m_{i}}}$$

where $m_{\mathrm{ij}}$ equals the fully de-conflated impact estimate for disease i. Eq. (16) is applied to all disease impacts such that $\sum_{j=1}^{n}\left({\mathrm{ep}}_{\mathrm{ji}}*m_{j}\right)$ captures all diseases in the model that satisfy the following conditions: (i) the diseases impact the same production characteristic as disease i, and (ii) the diseases are associated such that they have an inter-disease OR ≠ 1 with disease i. The process from (4), (5), (6), (7), (8), (9), (10), (11), (12), (13), (14), (15), (16) is then repeated for all other diseases in the model that satisfy respective versions of conditions (i) and (ii). A hypothetical three-disease example of this de-conflation process is detailed in Section 2 of this manuscript’s Supplementary File.

### 2.3 Productivity Gaps

Section 2.2 outlined a method for de-conflating disease impact estimates from the literature so that they may be aggregated without overlap. This section will outline the method used to estimate the yield, fertility, and mortality gaps due to endemic diseases and health conditions within a production system, aggregate and value those gaps to generate a total disease burden estimate, and attribute that burden to generate disease-specific loss estimates for endemic diseases and health conditions within a production system. For any production characteristic of that system, the observed mean value of that characteristic, or $\overset{®}{x}$, can be described using the following equation:

(17) $$\overset{®}{x}=\sum_{i=1}^{n}x_{i}*p_{i}$$

where $x_{i}$ equals the value of that characteristic for the i^th^ group, $p_{i}$ equals the proportion of the production system within the i^th^ group, and:

(18) $$x_{i}=x_{h}*r_{i}$$

where $x_{h}$ equals the value of that characteristic among disease-free animals and $r_{i}$ equals the fraction of that value realised (despite diseases and health conditions) by the i^th^ group. From (17), (18), it follows that:

(19) $$\overset{®}{x}=\sum_{i=1}^{n}x_{i}*p_{i}=\sum_{i=1}^{n}x_{h}*r_{i}*p_{i}=x_{h}*\sum_{i=1}^{n}r_{i}*p_{i}$$

where $\sum_{i=1}^{n}r_{i}*p_{i}$ equals the aggregate impact of the diseases being modelled:

(20) $$\sum_{i=1}^{n}r_{i}*p_{i}=1-\sum_{i=1}^{n}m_{\mathrm{ij}}*P\left(i\right)$$

Therefore, the value of a production characteristic among disease-free animals, or $x_{h}$, can be approximated using the following equation:

(21) $$x_{h}=\frac{\overset{®}{x}}{\sum_{i=1}^{n}r_{i}*p_{i}}=\frac{\overset{®}{x}}{1-\sum_{i=1}^{n}m_{\mathrm{ij}}*P\left(i\right)}$$

In other words, the disease-free value of a production characteristic can be approximated by the ratio of its observed mean to 1 less the sum of the products of the fully de-conflated disease impact estimates and their prevalence within that production system. This approach is comparable to Lichtenberg and Zilberman’s (1986) approach of modelling crop damage and loss adapted to the animal health perspective by Hennessy and Marsh (2021). The gap (difference) between the disease-free value and the observed mean value, or $x_{h}-\overset{®}{x}$, can then be attributed to the individual diseases according to the relative magnitudes of their de-conflated impacts using the following equation:

(22) $$g_{i}=\frac{m_{\mathrm{ij}}*P\left(i\right)}{m_{\mathrm{ij}}*P\left(i\right)+\sum_{k=1}^{n}\left[m_{\mathrm{kj}}*P\left(k\right)\right]}*(x_{h}-\overset{®}{x})$$

where $g_{i}$ equals the proportion of the productivity gap attributable to disease i and $m_{\mathrm{kj}}$ is the fully de-conflated impact estimate for disease k. This process can be repeated for any production characteristic that is impacted by diseases within the production system. A hypothetical three-disease example of this productivity gap attribution process is detailed in Section 3 of this manuscript’s Supplementary File.

### 2.4 Application to an example dairy cattle system

The final step is to assign a value to these productivity gaps and attribute the economic burden of the diseases being modelled. Because this process is unique to the production system being modelled, it is illustrated here using an example dairy cattle system with input values and supporting parameters from the UK. The impacts of the endemic diseases and health conditions on yield (milk production), fertility (calving interval), and mortality (culling risk) are considered. The costs of preventive measures (private veterinary expenditures) are also considered, however not from the perspective of productivity gaps, as will be described in Section 2.3.5. The economic characteristics of the UK dairy sector are described in Table 1.

**Table 1:** Economic characteristics of UK dairy cattle herds used in the illustration of the model.

Characteristic	Value	Unit	Reference
Farm-gate milk price	30.22^a^	£ /100 kg	AHDB (2021d)
Dairy cows	1850.00^b^	‘000 head	AHDB (2021c)
Head per herd	148.00^c^	head	AHDB (2021b)
Culling rate	27.00^d^	percent	Hanks and Kossaibati (2020)
Replacement price	1335.36^e^	£ /cow	AHDB (2021a)
Private veterinary expenditures	71.09^f^	£ /LSU	Gilbert and Rushton (2014)
Lifetime milk yield	13.00^d^	kg/cow/day	Hanks and Kossaibati (2020)
Milk yield	8737.00^d^	kg/cow/year	Hanks and Kossaibati (2020)
Calving interval	401.00^d^	days	Hanks and Kossaibati (2020)

a Average of January 2021 to August 2021 monthly average farm-gate price per litre excluding bonus using data from DEFRA. For simplicity, litres are assumed to be equivalent to kilograms.

b 2020 value compiled by AHDB using data from DEFRA, the Welsh Government, SEERAD, DAERA, and SCDA.

c 2018 value compiled by AHDB using data from DEFRA, DHI, the Welsh Government, SEERAD, DARD, and the Scottish Dairy Association.

d Median value reported. Assumed to be roughly equivalent the population mean given the sample size (n = 500), as proposed by Hozo et al. (2005).

e Weighted average of June 2021 prices for cows over/under 36 months sold. Values compiled by the AHDB using data from AHDB, LAA, and IAAS.

f Estimated value per livestock unit (LSU), where 1 cow = 1 LSU using data from 2011. Value converted to 2021 British pounds at an inflation rate of 19.3% (Inflation Tool, 2021a).

#### 2.4.1 Diseases and health conditions

The endemic diseases and health conditions included in the model and their cow-level prevalence in UK dairy herds are described in Table 2. It is important to note that there are 13 choose 2, or 132=78 possible disease pairs in the model. However, inter-disease ORs for only 19 pairs were obtained from the literature, described in Table 3, with all other ratios assumed to equal 1 (i.e., no association and therefore no impact conflation across disease pairs).

**Table 2:** Cow-level prevalence values for endemic diseases and conditions used to illustrate the model. Based on an example dairy cattle system with input values and supporting parameters from the UK. Diseases and conditions ranked in order of decreasing prevalence.

Disease/condition	Prevalence (proportion)	Reference
Lameness	0.30^a^	Afonso et al. (2020)
Mastitis	0.30^b^	Hanks and Kossaibati (2020)
Subclinical ketosis	0.22^c^	Suthar et al. (2013)
GIN disease	0.21^d^	Scott et al. (2019)
Neosporosis	0.15^e^	Reichel et al. (2013)
Metritis	0.10^c^	Suthar et al. (2013)
Fasciolosis	0.10^f^	May et al. (2019)
Cystic ovary	0.09^g^	Gröhn et al. (1995)
Milk fever	0.08	Esslemont and Kossaibati (1996)
Paratuberculosis	0.07	Woodbine et al. (2009)
Retained placenta	0.05^h^	Dubuc and Denis-Robichaud (2017)
Displaced abomasum	0.03^c^	Suthar et al. (2013)
Dystocia	0.02	Rumph and Faust (2006)

a Pooled prevalence.

b Median value reported. Assumed to be roughly equivalent the population mean given the sample size (500), as proposed by Hozo et al. (2005).

c 10-country average (Italy, Croatia, Hungary, Poland, Serbia, Slovenia, Portugal, Spain, Germany, and Turkey) used to approximate UK value.

d Disease caused by gastrointestinal nematodes (GIN); Study of samples collected from replacement heifers in 306 dairy herds from across Canada; GIN were detected in 20.9% of heifers.

e Meta-analysis estimate of Neospora caninum infections among UK dairy cattle.

f Weighted average of two estimates from German grassland herds in 2006; individual cow prevalence of 10.1% (97/963) in July and 9.1% (95/1036) in September.

g Mean value among 90 Friesian-Holstein dairy herds in England (average size of 152 cows) for cows calving during 12 months in 1992–1993.

h Study based on 126 commercial dairy herds from Québec, Canada.

**Table 3:** Inter-disease odds ratios (ORs) used to illustrate the model. Disease pairs ranked in order of decreasing OR.

Disease/condition	OR	Reference
Retained placenta: metritis	6.20	Gröhn et al. (1995)
Displaced abomasum: subclinical ketosis	4.25^a^	Gröhn et al. (1995)
Retained placenta: dystocia	4.10	Gröhn et al. (1990b)
Metritis: displaced abomasum	3.40^b^	Gröhn et al. (1989);Gröhn et al. (1990b)
Metritis: dystocia	3.20	Gröhn et al. (1990b)
Lameness: paratuberculosis	2.70	Smith and van Winden (2019)
Milk fever: displaced abomasum	2.50	Gröhn et al. (1989)
Mastitis: metritis	2.30^c^	Gröhn et al., 1990a, Gröhn et al., 1990b
Displaced abomasum: retained placenta	2.20	Gröhn et al. (1995)
Mastitis: displaced abomasum	2.10	Gröhn et al. (1990b)
Subclinical ketosis: milk fever	2.10	Gröhn et al. (1995)
Lameness: subclinical ketosis	2.01	Raboisson et al. (2014)
Mastitis: milk fever	1.90	Gröhn et al. (1990a)
Mastitis: paratuberculosis	1.89	Rossi et al. (2017)
Mastitis: cystic ovary	1.65^d^	Gröhn et al., 1990a, Gröhn et al., 1990b
Mastitis: subclinical ketosis	1.64	Raboisson et al. (2014)
Subclinical ketosis: cystic ovary	1.60	Gröhn et al. (1990b)
Metritis: subclinical ketosis	1.40	Dubuc et al. (2010)
Subclinical ketosis: retained placenta	1.20	Gröhn et al. (1989)

a Average of 4.0 for displaced abomasum being a predisposing condition for ketosis and 4.5 for ketosis being a predisposing condition for displaced abomasum.

b Average of 2.5 for metritis being a predisposing condition for displaced abomasum (Gröhn et al., 1989) and 4.3 for displaced abomasum being a predisposing condition for metritis (Gröhn et al., 1990b).

c Average of 1.6 for metritis being a predisposing condition for mastitis (Gröhn et al., 1990a) and 3.0 for mastitis being a predisposing condition for metritis (Gröhn et al., 1990b).

d Average of 1.8 for cystic ovary being a predisposing condition for mastitis (Gröhn et al., 1990a) and 1.5 for mastitis being a predisposing condition for cystic ovary (Gröhn et al., 1990b).

#### 2.4.2 Reduced yield (milk output)

The authors define milk output as the amount of milk produced per cow per year. The milk yield impacts of the diseases and health conditions modelled are described in Table 4. Using the methodology described in (4), (5), (6), (7), (8), (9), (10), (11), (12), (13), (14), (15), (16), these estimates are de-conflated according to the inter-disease ORs in Table 3. These de-conflated milk yield impact estimates are then used to estimate the milk yield gap attributable to disease using the methodology described in (17), (18), (19), (20), (21). The productivity gap (in kg of milk) is then valued using the farm-gate milk price in Table 1 and attributed to the diseases and health conditions in the model according to Eq. (22).

**Table 4:** Disease- and condition-specific total milk yield per lactation impact estimates used to illustrate the model. Based on an example dairy cattle system with input values and supporting parameters from the UK. Diseases and conditions ranked in order or decreasing impact.

Disease/condition	Impact (% decrease)	Reference
Retained placenta	7.38^a^	Dubuc et al. (2011)
Fasciolosis	7.33^b^	Mezo et al. (2011)
Paratuberculosis	5.90	McAloon et al. (2016)
Lameness	5.54^c^	Green et al. (2002)
Mastitis	4.57^d^	Seegers et al. (2003)
Neosporosis	4.20	Hernandez et al. (2001)
Dystocia	4.05^e^	Kaya et al. (2015)
Displaced abomasum	4.04^f^	Raboisson et al. (2014)
Metritis	3.95^g^	Giuliodori et al. (2013)
GIN disease	3.28^h^	Nødtvedt et al. (2002)
Subclinical ketosis	3.05^i^	Raboisson et al. (2014)
Milk fever	0.41^j^	Østergaard et al. (2003)
Cystic ovary	0.00	Gröhn et al. (1998)

a Projected effect of retained placenta was a reduction in milk yield of 753 kg/lactation. Given the observed mean milk production in the study was 10,210 kg/cow/lactation, this is equivalent to 7.4% of yield.

b Losses associated with F. hepatica estimated of 2.1 kg/cow/day equivalent to a yield loss of 7.3%, assuming a 305-day lactation and assuming a mean equivalent to the UK value (Table 1).

c Average losses per case of clinical lameness estimated at 360 kg/lactation equivalent to 5.5% of yield, assuming a mean equivalent to the UK value (Table 1).

d Meta-analysis resulted in an estimate of 375 kg/lactation lost equivalent to 4.6% of yield, assuming a mean equivalent to the UK value (Table 1).

e Study of Turkish Holstein cattle considering the entire 305-day milk yield. Cows with dystocia produced 219 kg less milk than cows with eutocia with a mean 305-day production of 5405.5 kg among all animals in the sample, equivalent to a 4.05% reduction in yield.

f Average value of the losses associated with low (300 kg/cow/lactation) and high (406 kg/cow/lactation) abomasal displacement equivalent to a yield loss of 4.0%, assuming a mean equivalent to the UK value (Table 1).

g Average value of the losses relative to metritis-free cows among clinical (411 kg/cow/90 DIM) and perpetual (280 kg/cow/90 DIM) equivalent to a yield loss of 4.0%, assuming a mean equivalent to the UK value (Table 1).

h Disease caused by gastrointestinal nematodes (GIN); Results of an intervention study; treatment with eprinomectin at calving was estimated to result in an increase 0.94 kg/cow/day equivalent to 3.3% of yield, assuming a mean equivalent to the UK value (Table 1).

i Meta-analysis resulted in an estimate of 340 kg/lactation lost equivalent to 3.1% of yield, assuming a mean equivalent to the UK value (Table 1).

j Default value used in the Østergaard et al. (2003) herd model SimHerd III was a 6% reduction for 21 days of the lactation due to the development of milk fever. Assuming a 305-day lactation, this is equivalent to 0.4% of yield.

#### 2.4.3 Reduced fertility (increased calving interval)

The authors define calving interval as the amount of time measured in days between the birth of a calf and birth of a subsequent calf from the same cow. Using an approach identical to that described for reduced yield (milk output), the fertility gap is estimated using the calving interval impact estimates in Table 5. The resulting gap (in days) is then valued as reduced output according to the average lifetime daily milk yield and the farm-gate price of milk from Table 1 and attributed to the diseases in the model. By valuing calving interval using the value of lifetime milk yield, which contains an implicit time component, the effects of delayed and/or shortened production windows are captured.

**Table 5:** Disease- and condition-specific calving interval impact estimates used to illustrate the model. Based on an example dairy cattle system with input values and supporting parameters from the UK. Diseases and conditions ranked in order of decreasing impact.

Disease/condition	Impact (% increase)	Reference
Lameness	12.47^a^	Hernandez et al. (2005)
Cystic ovary	11.26^b^	Laporte et al. (1994)
Neosporosis	7.21	Kamga-Waladjo et al. (2010)
Dystocia	6.96	Gaafar et al. (2011)
Paratuberculosis	5.79^c^	Ozsvari et al. (2020)
Metritis	4.74^d^	Fourichon et al. (2000)
Retained placenta	2.74^e^	Fourichon et al. (2000)
Subclinical ketosis	1.50^f^	Fourichon et al. (2000)
GIN disease	1.20^g^	Walsh et al. (1995)
Displaced abomasum	0.00	Fourichon et al. (2000)
Mastitis	0.00	Fourichon et al. (2000)
Milk fever	0.00	Fourichon et al. (2000)
Fasciolosis	0.00	Mezo et al. (2011);Howell et al. (2015)

a Median time to conception was 50 days longer among lame cows in the study, which is equivalent to a 12.5% increase in calving interval assuming a mean equivalent to the UK value (Table 1).

b Study indicated that cows with cystic ovarian disease (COD) had a mean calving interval of 425 days whereas cows without COD had a mean calving interval of 382 days, equivalent to an 11.3% increase.

c Test-positive cows conceived 23.2 days later than ELISA-negative cows equivalent to a 5.8% increase in calving interval, assuming a mean equivalent to the UK value (Table 1).

d Meta-analysis resulted in an estimated 19-day increase in time-to-conception, which is equivalent to a 2.7% increase in calving interval assuming a mean equivalent to the UK value (Table 1).

e Meta-analysis resulted in an estimated 11-day increase in time-to-conception, which is equivalent to a 2.7% increase in calving interval assuming a mean equivalent to the UK value (Table 1).

f A 6-day increase in time-to-conception was observed among cows with subclinical ketosis, which is equivalent to a 1.5% increase in calving interval, assuming a mean equivalent to the UK value (Table 1).

g Disease caused by gastrointestinal nematodes (GIN).

#### 2.4.4 Increased mortality (premature culling)

The authors define premature culling as a death or removal from the herd that would not have occurred in the absence of the diseases or health conditions being modelled. Once again, using an identical approach to that described for reduced yield (milk output) and reduced fertility (calving interval), the mortality gap is estimated and attributed using the culling hazard ratios described in Table 6. However, there is one additional step required prior to de-conflation and attribution: The culling hazard ratios must first be converted to excess culling probabilities, given the mean culling rate (Table 1), the cow-level prevalence of each disease and condition (Table 2), and the culling hazard ratios associated with each disease and condition (Table 6). To do so, (4), (5), (6), (7), (8), (9), (10), (11), (12), (13), (14) are applied to each disease and condition generating an annual excess probability of mortality equivalent to each culling hazard ratio. These excess mortality probabilities are then de-conflated as described for milk yield and fertility, with that excess mortality probability valued as a proportion of the replacement price (Table 1). Finally, these de-conflated mortality probabilities are converted back into culling hazard ratios so that they may be directly compared to the original, unadjusted hazard ratios obtained from the literature using the following equation:

(23) $${\mathrm{HR}}_{\mathrm{ij}}=\frac{{\mathrm{epm}}_{ij}*{\mathrm{HR}}_{i}}{{\mathrm{epm}}_{i}}$$

where ${\mathrm{HR}}_{\mathrm{ij}}$ is the de-conflated culling hazard ratio associated with disease i, ${\mathrm{HR}}_{i}$ is the raw culling hazard ratio associated with disease i as obtained from the literature, ${\mathrm{epm}}_{\mathrm{ij}}$ is the de-conflated excess probability of mortality associated with disease i equivalent to ${\mathrm{HR}}_{\mathrm{ij}}$, and ${\mathrm{epm}}_{i}$ is the excess probability of mortality associated with disease i equivalent to ${\mathrm{HR}}_{d_{i}}$.

**Table 6:** Disease- and condition-specific culling hazard ratios (HRs) and their equivalents in terms of annual excess probability of mortality used to illustrate the model. Based on an example dairy cattle system with input values and supporting parameters from the UK. Diseases and conditions ranked in order of decreasing HR.

Disease/condition	HR	Reference	Annual excess probability of mortality
Displaced abomasum	3.83	Sharifi et al. (2013)	0.31
Lameness	3.40	Sharifi et al. (2013)	0.25
Mastitis	2.78	Sharifi et al. (2013)	0.21
Milk fever	2.50^a^	Rajala-Schultz and Gröhn (1999b)	0.21
Paratuberculosis	2.40^b^	Hendrick et al. (2005)	0.20
Metritis	2.20	Rajala-Schultz and Gröhn (1999b)	0.17
Subclinical ketosis	2.10	Rajala-Schultz and Gröhn (1999b)	0.16
Dystocia	1.90	Rajala-Schultz and Gröhn (1999a)	0.14
Neosporosis	1.60	Thurmond and Hietala (1996)	0.10
Cystic ovary	1.00	Gröhn et al. (1998)	0.00
GIN disease	1.00^c^	Assumed	0.00
Fasciolosis	1.00^d^	Assumed	0.00
Retained placenta	1.00	Dubuc and Denis-Robichaud (2017)	0.00

a Value when the statistical model did not include milk yield (2.8 when yield was included in the model).

b Average of estimates for positive faecal culture (3.2), positive results of milk ELISA (2.3), and positive results of serum ELISA (1.7).

c Disease caused by gastrointestinal nematodes (GIN); No data available.

d No data available.

#### 2.4.5 Preventive measures (private veterinary expenditures)

Due to a lack of UK dairy cattle disease- and condition-specific veterinary expenditure impact estimates, the costs of preventive measures (private veterinary expenditures) cannot be estimated using the approach described for yield, fertility, and mortality. While several possible attribution methods were considered, such as assuming mean veterinary expenditures (Table 1) are allocated to diseases and conditions according to the costliness of those diseases and conditions, these methods require unjustifiable assumptions and add unnecessary complexity. Instead, mean per-cow private veterinary expenditures are directly added to the total estimated per-cow economic losses due to disease in a lump sum.

### 2.5 Comparison to other aggregation methods

The productivity gaps and the resulting economic losses due to endemic diseases estimated using the de-conflated impacts are compared to the productivity gaps and losses when using the unadjusted impacts directly from the literature. Also, the total losses, both de-conflated and unadjusted, are compared to the total losses as estimated when directly aggregated using the disease and condition-specific total economic loss estimates in Table 7.

**Table 7:** Disease- and condition-specific total economic impact estimates used in the direct linear aggregation of economic losses due to endemic diseases. Based on an example dairy cattle system with input values and supporting parameters from the UK. Diseases and conditions ranked in order of decreasing total impact.

Disease/condition	Total economic impact (£/cow/year)	Reference
Lameness	123.37^a^	Archer et al. (2010)
GIN disease	110.13^b^	Charlier et al. (2012)
Metritis	100.44^c^	Pérez-Báez et al. (2021)
Mastitis	98.84^d^	Hagnestam-Nielsen and Østergaard (2009)
Cystic ovary	66.01^e^	Kim et al. (2005)
Fasciolosis	33.29^f^	Schweizer et al. (2005)
Subclinical ketosis	27.14^g^	Mostert et al. (2018)
Paratuberculosis	26.83^h^	Rasmussen et al. (2021)
Dystocia	20.97^i^	Kaya et al. (2015)
Neosporosis	12.74^j^	Reichel et al. (2013)
Displaced abomasum	11.11^k^	Miller and Dorn (1990)
Retained placenta	10.99^l^	Joosten et al. (1988)
Milk fever	7.44^m^	Kossaibati and Esslemont (1997)
*Total losses*	*649.30*	*Calculated*

a 2010 estimate of 334.17 £ /case adjusted for inflation at 23.06% (Inflation Tool, 2021a) converted to per-cow impact assuming a prevalence of 0.30 (Table 2).

b Disease caused by gastrointestinal nematodes (GIN); 2010 estimate of 64 US$/cow benefit to whole herd anthelmintic application at calving adjusted for inflation at 20.62% (Inflation Tool, 2021c) and converted to GBP at 0.78 £ /US$ (World Bank, 2021).

c 2021 estimate of 513 US$/case converted to per-cow impact with a study prevalence of 0.251 (equivalent to 128.76 US$/cow) and converted to GBP at 0.78 £ /US$ (World Bank, 2021).

d 2009 estimate adjusted for inflation at 14.96% (Inflation Tool, 2021b) converted to US$ at 0.88 €/US$ and GBP at 0.78 £ /US$ (World Bank, 2021).

e 2005 estimate of 687 US$/case adjusted for inflation at 36.88% (Inflation Tool, 2021c), converted to GBP at 0.78 £ /US$ (World Bank, 2021), and converted to per-cow impact assuming a prevalence of 0.09 (Table 2).

f 2005 estimate of 299 €/case adjusted for inflation at 25.60% (Inflation Tool, 2021b), converted to US$ at 0.88 €/US$ and GBP at 0.78 £ /US$ (World Bank, 2021), and assuming a prevalence of 0.10 (Table 2).

g 2017 estimate of 130 €/case adjusted for inflation at 7.06% (Inflation Tool, 2021b), converted to US$ at 0.88 €/US$ and GBP at 0.78 £ /US$ (World Bank, 2021), and converted to per-cow impact assuming a prevalence of 0.22 (Table 2).

h 2021 estimate for Great Britain of 34.40 US$/cow/year converted to GBP at 0.78 £ /US$ (World Bank, 2021).

i 2015 estimate of 24.24 US$/cow in any parity adjusted for inflation at 10.93% (Inflation Tool, 2021c) and converted to GBP at 0.78 £ /US$ (World Bank, 2021).

j 2013 estimate of 1800 US$/farm adjusted for inflation at 13.45% (Inflation Tool, 2021c), converted to GBP at 0.78 £ /US$ (World Bank, 2021), and converted to per-cow impact assuming 125 cows/farm in 2013 (AHDB, 2021b).

k 1990 estimate of 172.40 US$/cow-year in total disease losses with 4% due to displaced abomasum adjusted for inflation at 106.56% inflation (Inflation Tool, 2021c) and converted to GBP at 0.78 £ /US$ (World Bank, 2021).

l 1988 estimate of 471 £ /100-cow-year adjusted for inflation at 133.26% (Inflation Tool, 2021a).

m 1997 estimate of 59 £ /case adjusted for inflation at 57.64% (Inflation Tool, 2021a) and converted to per-cow impact assuming a prevalence of 0.22 (Table 2).

### 2.6 Sensitivity analyses

Using Palisade’s @ Risk v.8.2.0 software (Palisade, 2021), 50,000-iteration Monte Carlo simulations are used to test the sensitivity of the estimated total economic losses to variations in the model’s input variables. All variables are assumed to have (generalised) beta distributions ($a_{1}=2$ and $a_{2}=2$) with varying boundaries to introduce stochasticity to the proportions and probabilities used as input values. Specifically, prevalence values are bounded by 0 and 1 and thus require the standard two-parameter beta distribution. The additional two parameters for the four-parameter generalised beta distribution were introduced when values were bounded by values other than 0 and 1. Thus, the economic characteristics of the UK dairy sector are assumed to be bounded by 10% of their static values, the fertility impact percentages are bounded by 0 and 20, and inter-disease ORs and culling hazard ratios are bounded by 0 and 5. These boundaries were arbitrarily selected based on author judgement solely to demonstrate the ability to introduce stochasticity to the model and the potential usefulness of sensitivity analyses within this methodological framework.

## 3 Results

### 3.1 De-conflation

The de-conflation process generated adjusted impact estimates (Table 8) that can be aggregated and used to estimate the total economic losses due to endemic diseases and conditions. When compared to the unadjusted values directly from the literature using an example dairy cattle system (Table 4, Table 5, Table 6), de-conflation reduced impact estimates by an average of 25% among those estimates adjusted and by 17% overall. The most pronounced adjustments were to the overall impacts (average of yield, fertility, and mortality impacts) of subclinical ketosis (39% reduction), displaced abomasum (31% reduction), paratuberculosis (31% reduction), and metritis (28% reduction), which were the diseases with the greatest inter-diseases ORs (Table 3). Fasciolosis, GIN disease, and neosporosis were unaffected by the de-conflation process as all inter-disease ORs relating them to other diseases and conditions were assumed to equal 1 in the model. Despite associations with mastitis and subclinical ketosis, the overall impact of cystic ovary, which consists entirely of a fertility impact in the model, was not significantly affected by de-conflation because mastitis was not considered to have a fertility impact and the fertility impact of subclinical ketosis was relatively small (a 1.5% increase in calving interval, as described in Table 5).

**Table 8:** Disease and health condition impact estimates after being de-conflated for the impacts of associated diseases and conditions, including culling hazard ratios and their equivalent (≡) annual excess probability of mortality. Based on an example dairy cattle system with input values and supporting parameters from the UK. Diseases and health conditions in alphabetical order.

Disease/condition	De-conflated impact estimates		
	Output (% reduction in milk yield)	Fertility (% increase in calving interval)	Mortality (culling hazard ratio ≡ annual excess probability of mortality)
Cystic ovary	0.00	11.13	1.00 ≡ 0.00
Displaced abomasum	2.49	0.00	2.68 ≡ 0.31
Dystocia	2.96	6.04	1.60 ≡ 0.14
GIN disease^a^	3.28	1.20	1.00 ≡ 0.00
Fasciolosis	7.33	0.00	1.00 ≡ 0.00
Lameness	4.76	11.90	3.00 ≡ 0.22
Mastitis	3.72	0.00	2.22 ≡ 0.17
Metritis	2.39	4.10	1.55 ≡ 0.17
Milk fever	0.09	0.00	1.89 ≡ 0.16
Neosporosis	4.20	7.21	1.60 ≡ 0.10
Paratuberculosis	4.42	3.83	1.64 ≡ 0.13
Retained placenta	6.09	1.69	1.00 ≡ 0.00
Subclinical ketosis	2.65	0.54	1.29 ≡ 0.10

a Disease caused by gastrointestinal nematodes (GIN).

### 3.2 Productivity gaps

The estimated potential values of key production characteristics and the resulting productivity gaps due to endemic diseases in the example dairy cattle system are described in Table 9. Using de-conflated impact estimates, potential mean annual milk yield was estimated to be 9306 kg/cow, equivalent to a gap of 569 kg/cow from the current UK mean with a value of £ 172 cow/year. The potential calving interval was estimated to be 375 days/cow, equivalent to a gap of 26 days/cow valued at £ 102 cow/year. The potential culling rate was estimated to be 23% of cows per year, equivalent to a gap of 4% valued at £ 59 cow/year. When aggregated using unadjusted values, the yield, fertility, and mortality gaps were 21%, 9%, and 21% greater, respectively. The total value of these productivity gaps increased from the de-conflated value of £ 333 cow/year to the unadjusted value of £ 390 cow/year, equivalent to a 17% increase.

**Table 9:** Estimated per-cow productivity potential (in the absence of endemic diseases and health conditions) and resulting productivity gaps (potential less current mean) valued in GBP per cow/year. Based on an example dairy cattle system with input values and supporting parameters from the UK.

	Yield – Milk output kg/cow/year	Fertility – Calving interval (days/cow)	Mortality – Culling rate (% cows/year)	Total value *(£/cow/year)*
Mean	8737.00^a^	401.00^a^	27.00^a^	
	***De-conflated values***			
Potential	9306.32	375.09	22.56	
Gap (potential less mean)	569.32	25.91	4.44	
*Gap value (£/cow/year)*	*172.05*	*101.79*	*59.27*	*333.12*
	***Unadjusted values***			
Potential	9424.44	372.79	21.63	
Gap (potential less mean)	687.44	28.21	5.37	
*Gap value (£/cow/year)*	*207.75*	*110.83*	*71.72*	*390.29*

a Mean value as reported in Table 1.

### 3.3 Economic losses

The results of the attribution of the productivity gap values in Table 9 are presented as disease- and condition-specific economic losses in Table 10. The costliest disease/condition modelled was lameness, with estimated annual per-cow losses of £ 113 (de-conflated) and £ 124 (unadjusted). The next costliest disease modelled was mastitis with estimated annual per-cow losses of £ 47 (de-conflated) and £ 58 (unadjusted), with neosporosis (de-conflated and unadjusted annual per-cow losses of £38), subclinical ketosis with annual per-cow losses of £ 24 (de-conflated) and £ 39 (unadjusted), and fasciolosis (de-conflated and unadjusted annual per-cow losses of £20) also being significant contributors to total losses. When private veterinary expenditures were included in the total losses, estimated annual per-cow losses totalled £ 404 when de-conflated and £ 461 when unadjusted. When directly aggregated using disease-specific total loss estimates from the literature (Table 7), annual losses increased by 61% to £ 649/cow relative to the estimated de-conflated total losses including veterinary expenditures and increased by 41% relative to the estimated unadjusted total losses including veterinary expenditures.

**Table 10:** Estimated economic losses due to endemic diseases and health conditions using both de-conflated impact estimates and unadjusted endemic disease impact estimates. Based on an example dairy cattle system with input values and supporting parameters from the UK. Diseases and conditions in alphabetical order.

Disease/condition	*Disease-specific economic losses (£/year/cow) De-conflated*				*Disease-specific economic losses (£/year/cow) Unadjusted*			
	Yield	Fertility	Mortality	*Total*	Yield	Fertility	Mortality	*Total*
Cystic ovary	0.00	15.09	0.00	*15.09*	0.00	15.17	0.00	*15.17*
Displaced abomasum	2.10	0.00	1.99	*4.08*	3.66	0.00	2.72	*6.38*
Dystocia	1.66	1.78	0.72	*4.16*	2.31	2.04	0.82	*5.17*
Fasciolosis	19.79	0.00	0.00	*19.79*	20.04	0.00	0.00	*20.04*
GIN disease^a^	19.28	3.69	0.00	*22.97*	19.52	3.66	0.00	*23.19*
Lameness	40.09	52.56	20.39	*113.04*	47.32	54.78	22.15	*124.26*
Mastitis	31.37	0.00	15.34	*46.72*	39.05	0.00	18.46	*57.51*
Metritis	6.45	5.80	3.55	*15.79*	10.80	6.66	4.83	*22.29*
Milk fever	0.20	0.00	3.61	*3.82*	0.91	0.00	4.58	*5.48*
Neosporosis	17.71	15.93	4.47	*38.11*	17.93	15.83	4.28	*38.05*
Paratuberculosis	8.72	3.97	2.83	*15.53*	11.76	5.93	3.97	*21.66*
Retained placenta	8.39	1.22	0.00	*9.61*	10.29	1.97	0.00	*12.26*
Subclinical ketosis	16.29	1.76	6.37	*24.41*	24.15	4.78	9.91	*38.84*
*Total losses*	*172.05*	*101.79*	*59.27*	*333.12*	*207.75*	*110.83*	*71.72*	*390.29*
*Total + veterinary costs*^b^				*404.21*				*461.38*

a Disease caused by gastrointestinal nematodes (GIN).

b Mean annual private veterinary expenditures per cow per year (Table 1) added to the total losses due to the endemic diseases and conditions modelled.

### 3.4 Sensitivity analyses

**Fig. 1:**
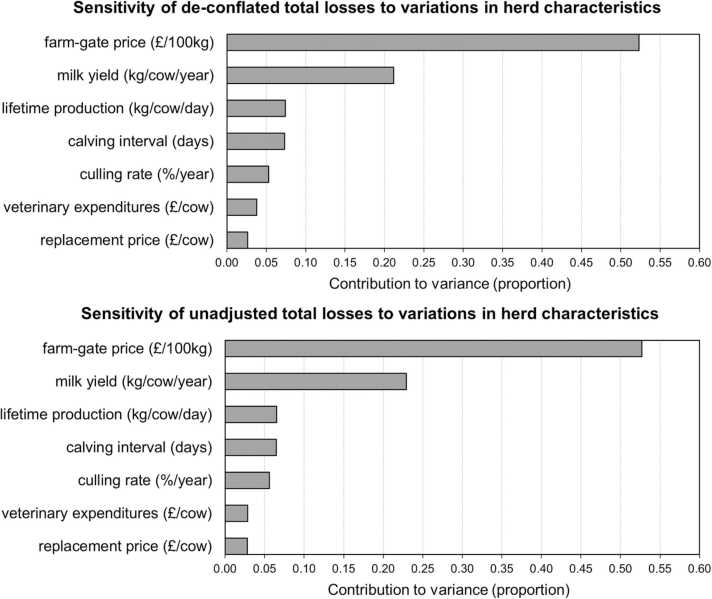
Sensitivity of total estimated losses due to endemic diseases and health conditions to variations in the values of herd characteristics. Herd characteristics are ranked according to the proportion of the total variance in total losses contributed by variations in the herd characteristics, in descending order. Results from 50,000-iteration Monte Carlo simulations of an example dairy cattle system using input values and supporting parameters from the UK.

**Fig. 2:**
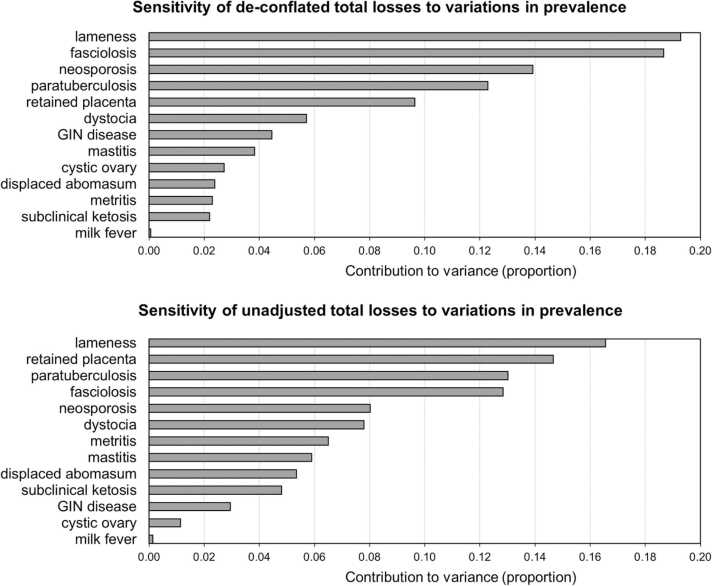
Sensitivity of total estimated losses due to endemic diseases and health conditions to variations in the prevalence of those diseases and conditions. Diseases and conditions are ranked according to the proportion of the total variance in total losses contributed by variations in their prevalence, in descending order. Results from 50,000-iteration Monte Carlo simulations of an example dairy cattle system using input values and supporting parameters from the UK.

**Fig. 3:**
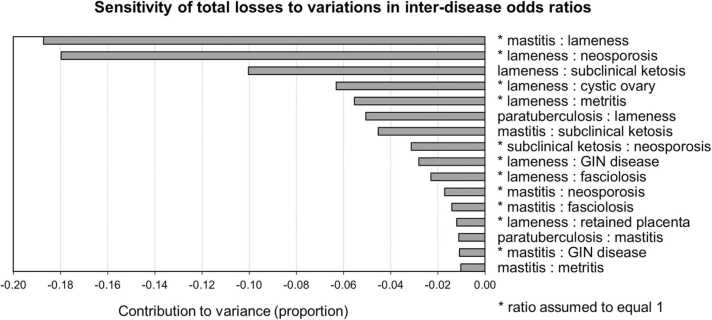
Sensitivity of total estimated losses due to endemic diseases and health conditions to variations in the values of inter-disease odds ratios (ORs). Pairwise ORs are ranked according to the proportion of the total variance in total losses contributed by variations in their magnitude, in descending order. Results from 50,000-iteration Monte Carlo simulations of an example dairy cattle system using input values and supporting parameters from the UK. An inter-disease odds ratio labelled with an asterisk indicates that outside of the Monte Carlo sensitivity analysis, its value was set to 1.

The results of the 50,000-iteration Monte Carlo sensitivity analyses are presented in Figs. 1 through 3. When considering basic herd characteristics, whether de-conflated or unadjusted, total estimated losses per cow were most sensitive to variations in the farm-gate price of milk and were similarly (across de-conflated and unadjusted estimates) sensitive to variations in other herd characteristics (Fig. 1). Both de-conflated and unadjusted estimated total losses were most sensitive to variations in the prevalence of lameness in the herd (Fig. 2). However, while the second largest contributor to the variance of de-conflated total losses was variation in the prevalence of fasciolosis, when unadjusted, the second largest contributor was variation in the prevalence of retained placenta. Both de-conflated and unadjusted estimates were similarly sensitive to variations in the prevalence of paratuberculosis and neosporosis. The two most impactful inter-disease ORs, by a large margin, were the mastitis-lameness and lameness-neosporosis associations, which contributed proportions of − 0.19 and − 0.18 to the variance, respectively (Fig. 3). However, both associations were assumed to be nonexistent in the model (inter-disease ORs assumed to equal 1). The most impactful associations that contributed to the de-conflation process (modelled with inter-disease ORs not equal to 1), were lameness-subclinical ketosis, paratuberculosis-lameness, and mastitis-subclinical ketosis, which contributed proportions of − 0.10, − 0.05, and − 0.04 to the variance of estimated losses, respectively. All inter-disease ORs were negatively related to total losses indicating that as these associations increase in magnitude, the effect of de-conflation also increases, and therefore estimated de-conflated total losses decrease.

## 4 Discussion

Once applied to an example dairy cattle system with input values and supporting parameters from the UK, it was demonstrated that the estimated total economic burden due to an array of diseases and conditions endemic to a production system varies depending on the aggregation method used. Direct linear aggregation of economic loss estimates resulted in greater total losses compared to losses estimated using the approach of valuing productivity gaps based on disease-specific milk yield, fertility, and mortality impacts, even with private veterinary expenditures included. It was also demonstrated that estimated total losses are further reduced when productivity gaps are calculated with consideration for statistical associations between diseases (inter-disease ORs). The results identified impactful disease associations, potentially impactful associations that may warrant further investigation, and suggest that from a disease control policy perspective, the costliness of a disease may not always be the best indicator of the investment its control warrants; the costliness rankings were inconsistent across approaches and total losses were found to be surprisingly sensitive to variations in the prevalence of relatively uncostly diseases. To the authors’ knowledge, this was the first attempt to estimate the aggregate economic burden due to diseases endemic to UK dairy cattle since Bennett et al. (1999) estimated the annual regional losses associated with bovine diarrhoea virus, fasciolosis, lameness, leptospirosis, and mastitis in mainland UK (England, Scotland, and Wales) dairy herds.

When directly aggregated using per-cow economic loss estimates from the literature, the total annual economic burden due to diseases and health conditions endemic to UK dairy cattle was calculated to be £ 649 per cow (Table 7). At the herd level, this is equivalent to annual losses of £ 96,000. While it is impossible, at this stage of research, to determine if direct linear aggregation generally results in an overestimation of the total burden of an array of diseases, when these losses are compared to the average herd-level gross milk revenue in 2021 among UK dairy herds, £ 391,000 (the product of head per holding, annual production, and farm-gate price from Table 1), direct aggregation suggests that 25% of gross milk revenue is lost due to endemic diseases, which seems implausibly large. Still, this estimate is useful in that it provides a benchmark for comparison of the results of the more refined aggregation approaches used in this study: 1) estimation and attribution of productivity gaps using disease- and condition-specific yield, fertility, and mortality impact estimates from the literature (an unadjusted estimate); 2) estimation and attribution of productivity gaps with consideration for inter-disease associations (a de-conflated estimate).

Without de-conflation, unadjusted annual per-cow losses due to endemic diseases including private veterinary expenditures were estimated to be £ 461 (Table 10), equivalent to annual herd-level losses of £ 68,000, a 29% reduction from the directly aggregated estimate. When de-conflated to account for inter-disease associations, estimated annual per-cow losses including private veterinary expenditures decreased to £ 404 (Table 10), equivalent to herd-level losses of £ 60,000, a 38% reduction. While both estimates are still equivalent to significant proportions of herd-level gross milk income (17% and 15%, respectively), these results suggest, at the very least, that estimating and attributing productivity gaps provides an alternative to direct linear aggregation and generates seemingly more plausible results. Directly aggregated economic loss estimates for an array of endemic diseases are likely to overestimate the total economic burden, and by restricting potential losses based on the impacts of those diseases and conditions on key production variables and the observed mean values of those variables, the potential for overestimation is reduced. The results also suggest that ignoring inter-disease associations or disease overlap across related diseases contributes, in part, to that potential for overestimation.

These different aggregation approaches generate not just different estimates of the total burden, but also different rankings of the costliness of the diseases and conditions being modelled (Table 7, Table 10). For example, while all three methods suggested lameness to be the costliest endemic condition in UK dairy herds by far, direct linear aggregation was the only approach that suggested GIN disease and metritis are more costly than mastitis. There were other disagreements across the results of the three aggregation approaches, most notably neosporosis being ranked much lower in terms of costliness using direct linear aggregation and cystic ovary being ranked much lower using both the unadjusted and de-conflated productivity gap aggregation approaches. However, the particulars of these disagreements are not as important as the fact that there were disagreements; if costliness is the primary metric used to determine a hierarchy of disease control priorities and formulate animal health policy, then important nuances may be missed when solely comparing economic loss estimates like those used in the direct linear aggregation approach. This study suggests that disease control priorities may be more accurately determined by considering multiple endemic diseases and conditions simultaneously, the impacts of those diseases on key production variables, and the associations and relationships between those diseases and conditions within the same methodological framework. This suggestion is further reinforced by examining the sensitivity of estimated total losses to variations in the prevalence of diseases and conditions using both the unadjusted and de-conflated productivity gap approaches (Fig. 2).

With both the unadjusted and de-conflated approaches, total economic losses were most sensitive to variations in the prevalence of lameness. However, much as the costliness rankings were inconsistent across approaches, so too were the rankings in terms of sensitivity across the productivity gap approaches. For example, when unadjusted, variations in the prevalence of fasciolosis contributed a proportion of 0.13 to the variance of total economic losses (4th in the rankings). Once de-conflated, variations in its prevalence contributed a proportion of 0.19 to the variance in losses (2nd in the rankings), comparable to the proportion of 0.20 contributed by variations in the prevalence of lameness. Metritis, on the other hand, contributed a proportion of 0.06 when unadjusted (7th in the rankings), but once de-conflated contributed a proportion of just 0.02 (11th in the rankings). Similarly, the contribution to variance due to variations in the prevalence of retained placenta decreased from 0.15 (2nd in the rankings) to 0.10 (5th in the rankings) once de-conflated. The de-conflation process negatively impacted the contribution to variance, or in other words, the significance, of diseases that were most strongly associated with other diseases in the model (e.g., metritis, subclinical ketosis, retained placenta, paratuberculosis, and mastitis). At the same time, the process positively impacted the significance of diseases that were not related to other diseases in the model (e.g., neosporosis, fasciolosis, and GIN disease) because these unrelated diseases were not affected by the de-conflation process and variations in their prevalence were now relatively more impactful. These results highlight the effect of incorporating inter-disease associations when aggregating disease impacts and the importance of considering more than just relative costliness when forming a disease priority hierarchy.

Relative costliness alone only informs on the status quo since it is an estimate based primarily on current prices and current prevalence. However, by also examining the sensitivity of total losses to variations in prevalence, inferences can be made about losses given a change in the prevalence situation. Given that controlling the prevalence of disease is among the primary aims of animal health policy, the sensitivity of total losses to variations in prevalence may perhaps lead to more accurate hierarchical rankings of control priorities. This is particularly important for infectious diseases and conditions where affected animals may positively contribute to the infectious load within a herd, potentially leading to prevalence increases or epidemics. The results suggest that the two measures, costliness of a disease and sensitivity of total losses to variations in that disease’s prevalence, do not always align; when comparing the costliness of the diseases and conditions (Table 10) to the sensitivity of total losses to variations in prevalence (Fig. 2), whether unadjusted or de-conflated, the rankings differ. Once again, all aggregation methods and sensitivity analyses suggested lameness to be the costliest disease and variations in its prevalence the most impactful. However, neither the unadjusted nor the de-conflated disease-specific loss estimates align with the corresponding estimated costliness of the diseases resulting from each approach. The top panel of Fig. 2 suggests that using the de-conflated approach, the impacts of variations in the prevalence of fasciolosis, neosporosis, and paratuberculosis are comparable to the impact of variations in the prevalence of lameness. However, this same approach also suggests that fasciolosis, neosporosis, and paratuberculosis are only the 6th, 3rd, and 8th costliest diseases, respectively (Table 10). Conversely, mastitis, which is the 2nd costliest disease is ranked 8th in terms of how variations in its prevalence impact estimated total losses. While this may seem counterintuitive, this can be explained by the relative impacts of these diseases on the sources of losses in the model (yield (Table 4), fertility (Table 5), and mortality (Table 6)) their prevalence values (Table 2), and their associations with other diseases (Table 3).

Fasciolosis and paratuberculosis both have below-average prevalence values resulting in low rankings in terms of costliness (Table 10). At the same time, their above-average yield impacts, the greatest contributor to losses in the model (43% of total losses with veterinary expenditures included), and above-average fertility and mortality impacts for paratuberculosis cause variations in the prevalence of paratuberculosis to be more impactful than variations in the prevalence of fasciolosis. However, due to the associations between paratuberculosis and mastitis and paratuberculosis and lameness, once de-conflated for the impacts of these associated diseases, variations in the prevalence of paratuberculosis become less impactful than variations in the prevalence of fasciolosis, which despite having no fertility or mortality impacts, has no disease associations in the model and is therefore unaffected by de-conflation. Neosporosis, which has on-average prevalence and yield impact, above-average fertility impact, and slightly below-average mortality impact, is highly ranked in terms of costliness. But, like fasciolosis, neosporosis shares no associations in the model and is unaffected by de-conflation, resulting in variations in its prevalence being comparably impactful whether de-conflated or not. Mastitis, on the other hand, has the second highest prevalence value (0.30), but has just an on-average yield impact, no fertility impact, and an on-average mortality impact in the model. This combination leads to a high ranking in terms of costliness, but due to slightly below-average overall impact and the disease’s associations with paratuberculosis, subclinical ketosis, milk fever, displaced abomasum, metritis, and cystic ovary, once de-conflated, total losses are only minorly impacted by variations in its prevalence.

These two measures of economic importance paint very different pictures in terms of disease control priorities, and the contrasts between disease-specific economic costliness and the impact of variations in prevalence highlight the importance of disease associations when modelling an array of diseases. Fig. 3 identifies several key inter-disease ORs, most of which are assumed to equal 1 in the model and are therefore only impactful within the framework of the sensitivity analyses. However, some of these null associations may have some physiological or biological basis and may therefore warrant further investigation. Also, the same figure illustrates the previously described mechanism whereby the magnitude of de-conflation, and therefore the magnitude of the inter-disease ORs, are inversely related to estimated total losses. The approach assumes that the stronger the association, the more overlap, the more excess probability of disease across the pair of diseases, and the more impact conflation must be adjusted for.

The importance of disease associations in the model is in some ways a limitation. As previously mentioned, this is not a mechanistic model because causal associations and random, statistical associations are treated as the same due the aforementioned “snapshot” assumption. However, this assumption results in a slightly misleading result whereby the stronger the causal relationship between two diseases, the greater the impact of de-conflation. While this is completely logical for statistical associations between diseases where a stronger association implies more disease overlap, this assumption is only valid for causal relationships that are expressed within the timeframe of the snapshot being modelled. In this case, one year. For long-term causal relationships or causal relationships that manifest across age groups, this assumption may be too rigid. To determine the impact of this assumption, the de-conflation process was repeated with four potentially conflicting causal associations (displaced abomasum-subclinical ketosis, mastitis-metritis, metritis-displaced abomasum, and mastitis-cystic ovary), and three inter-lactational associations from Gröhn et al. (1995) (retained placenta-metritis, displaced abomasum-retained placenta, and subclinical ketosis-milk fever), assumed to equal 1. The results were similar, with estimated total annual economic losses including private veterinary expenditures equal to £ 417 per cow, an increase of only 3% from the fully de-conflated result. See Table A1 of the Appendix for more details.

It is also important to note that while the de-conflation approach described captures economic importance both in terms of costliness and sensitivity of losses to variations in prevalence, it does not capture causal information; this approach looks only at economic effects and not economic causes. Modelling causal relationships would require a different approach akin to path analysis, whereby the economic impacts of diseases and conditions down the causal path would be attributed to the predisposing disease or condition. For example, if a single disease or condition were the cause of all other diseases and conditions in the model, then the entire economic burden would be attributed to that single disease.

Whatever the aggregation method used, the accuracy of the estimated total losses depends heavily on the accuracy, rigour, and generalisability of the studies from which the input values, particularly prevalence, disease impacts, and inter-disease ORs, are sourced. To mitigate the impact of potential flaws in the selected input values, in future applications of the described approach the authors will aim to use systematic reviews and meta-analyses to populate the model. The authors will also add stochasticity according to reported confidence intervals and distributions of input variables through Monte Carlo simulations. These additional levels of complexity will generate a range of statistically significant disease burden estimates, as opposed to the point estimates presented herein, that will reflect the uncertainty inherent to modelling efforts such as this one that combine data from varied, potentially unreliable sources. The authors also anticipate that when the described method is applied in data-scarce environments, despite systematic reviews and meta-analyses, generalisations, assumptions, and data simulations will likely be needed to fully populate the model.

Additionally, the de-conflation method as described assumes that all disease interactions are additive and in the same direction. In other words, if an animal has both diseases i and k and these diseases are positively associated, the model assumes that the excess probabilities of disease i among animal with disease k and vice versa result in overestimations of the individual disease impacts and de-conflation therefore reduces the impacts of each disease. However, it is possible that not only are some disease impacts non-additive (e.g., non-linear, multiplicative, etc.), but also that some diseases or conditions may dampen the effects of other diseases or conditions. For example, the effects of coinfection by multiple parasitic species may have varied magnitudes and directions (Graham, 2008), and by disentangling de-conflation from the disease interaction aspects of the proposed method, the authors intend to allow for more biologically accurate modelling of coinfections and comorbidities (i.e., synergistic and antagonistic interactions) in future applications. Therefore, it is important to recognise that the UK dairy cattle disease burden estimates presented are, at this stage, relatively crude “best guesses” and serve primarily as proof-of-concept, illustrating the model’s functionality. Until the model’s input variables have been systematically reviewed and subjected to meta-analysis, uncertainty surrounding the model’s input variables has been captured, and, where appropriate, more nuanced disease interaction mechanisms have been introduced to the model, the disease burden estimates presented should be interpreted with caution. The authors suggest that to further develop this methodology, and if other researchers wish to use the described approach to account for disease overlap in their economic and epidemiologic studies, prevalence values, inter-disease odds ratios across disease pairs, and the impacts on yield, fertility, and mortality associated with the diseases and conditions observed in the study sample, concomitant or otherwise, should be reported. Also, while currently in Microsoft Excel (Microsoft, 2021) spreadsheet form, the authors will explore programming the model, along with refinements to capture uncertainty surrounding input variables and varied disease interaction mechanisms, into a package for R, a free software environment for statistical computing and graphics (The R Foundation, 2021).

Finally, the approach described is only applicable to endemic diseases, or diseases and conditions that coexistent on a quasi-permanent basis within a livestock production system, and that directly impact productivity. This approach does not capture economic losses attributable to health issues such as malnutrition, some injuries, or predation, which would form much of the remainder of the complete AHLE, as described in the Introduction. Several studies aimed at better understanding the magnitude of this comprehensive envelope are underway within GBADs, and research into possible ways of estimating and attributing the remainder of the AHLE is ongoing. It is also important to note that the approach used in this study fails to capture the economic losses associated with sporadic, heterogeneously distributed epidemic diseases that result in periodic mandatory culling such as bovine tuberculosis (bTB). Between March 2020 and March 2021, approximately 9 million bTB cattle (beef and dairy) tests were administered, resulting in mandatory culls of 39,000 cattle in Great Britain alone (DEFRA, 2021). It has been estimated that this testing and culling programme results in mean costs of over £ 22,000 per impacted cattle herd within high risk and edge areas of England and and high and intermediate TB areas of Wales (Barnes et al., 2020). However, these areas are unlikely to be representative of the UK as a whole, and although associations between reduced milk production and bTB infection have been observed (Hernandez and Baca, 1998, Rahman and Samad, 2008), it has also been suggested that less productive dairy cattle may be more susceptible to bTB as opposed to bTB resulting in reduced production (Boland et al., 2010). Regardless of its productivity impacts, bTB is listed by the World Organisation for Animal Health (OIE) and must be reported according to the OIE’s Terrestrial Animal Health Code (OIE, 2021) largely due to its zoonotic potential. Therefore, economic losses due to bTB may be primarily attributable to control programmes aimed at preventing catastrophic losses due to potential trade restrictions (Gordon, 2008) as opposed to the disease’s direct negative impacts on productivity. Despite a significant economic burden resulting from bTB within the UK dairy sector, the disease does not fit within the framework of this endemic disease model and was thus excluded from the analysis.

## 5 Conclusions

This study explored three approaches to aggregating the per-animal annual economic impact of an array of endemic diseases: 1) direct linear aggregation of economic losses, 2) estimation and attribution of productivity gaps using disease- and condition-specific yield, fertility, and mortality impact estimates from the literature, and 3) estimation and attribution of productivity gaps with consideration for inter-disease associations. These approaches were then compared using an example dairy cattle system with input values and supporting parameters from the UK, resulting in estimated total annual losses of £ 404 per cow, equivalent to herd-level losses of £ 60,000/year. Unadjusted productivity gap aggregation suggested losses 14% greater, while direct linear aggregation suggested losses 61% greater. Reduced milk yield accounted for approximately 40% of de-conflated losses, while reduced fertility, increased mortality, and private veterinary expenditures accounted for 25%, 15%, and 20%, respectively. Although lameness was identified as the costliest condition (28% of total losses), variations in the prevalence of fasciolosis, neosporosis, and paratuberculosis (only a combined 20% of total losses) were nearly as impactful individually as variations in the prevalence of lameness. Associations between lameness and subclinical ketosis, paratuberculosis and lameness, and mastitis and subclinical ketosis were identified as particularly impactful. Once refined and widely available, the model will flexibly allow for endemic disease impact estimates in any livestock system and region to be aggregated without double counting. Since the results can be updated as new prevalence, impact, and disease association data become available, the model will also provide an alternative tool to rank disease control priorities for the formulation of animal health policy.

## Funding

This research is on behalf of the Global Burden of Animal Diseases (GBADs) Programme which is led by the University of Liverpool, United Kingdom and the World Organisation for Animal Health, France (OIE). This research is supported through the Grant Agreement Investment ID INV-005366 with the 10.13039/100000865Bill & Melinda Gates Foundation, USA and the UK Foreign, Commonwealth and Development Office, United Kingdom (FCDO). GBADs case studies receive additional funding from the following: European Commission, Belgium, Australian Centre for International Agricultural Research, Australia (10.13039/501100000974ACIAR), Brooke Foundation, United Kingdom, and the Food and Agriculture Organisation of the United Nations, Italy (FAO).

## Author contributions

AS, PT, and MB conceived of the research. PR conceived of, developed, and programmed the model, performed the simulations and computations, and led the writing of the manuscript. PT, AS, VM, and MB verified the methodology and validity of the results. All authors discussed the results and reviewed the final manuscript.

## Figures and Tables

**Table A1:** Estimated economic losses due to endemic diseases and health conditions in dairy cattle using de-conflated impact estimates. Calculated with odds ratios of potentially conflicting causal associations (displaced abomasum-subclinical ketosis, mastitis-metritis, metritis-displaced abomasum, and mastitis-cystic ovary) and inter-lactational associations from Gröhn et al. (1995) (retained placenta-metritis, displaced abomasum-retained placenta, and subclinical ketosis-milk fever) assumed to equal 1. Based on an example dairy cattle system with input values and supporting parameters from the UK. Diseases and conditions in alphabetical order.

Disease/condition	Economic losses (£/cow/year) – De-conflated			
	Yield	Fertility	Mortality	*Total*
Cystic ovary	0.00	15.08	0.00	*15.08*
Displaced abomasum	3.04	0.00	2.38	*5.42*
Dystocia	1.67	1.78	0.71	*4.16*
Fasciolosis	19.85	0.00	0.00	*19.85*
GIN disease^a^	19.34	3.68	0.00	*23.02*
Lameness	40.22	52.53	20.19	*112.94*
Mastitis	33.33	0.00	16.03	*49.36*
Metritis	9.76	6.26	4.60	*20.61*
Milk fever	0.30	0.00	3.91	*4.21*
Neosporosis	17.76	15.92	4.43	*38.11*
Paratuberculosis	8.75	3.97	2.81	*15.53*
Retained placenta	9.76	1.73	0.00	*11.48*
Subclinical ketosis	17.12	1.76	7.13	*26.01*
*Total losses*	*180.89*	*102.70*	*62.19*	*345.78*
*Total + veterinary costs*^b^				*416.87*

a Disease caused by gastrointestinal nematodes (GIN).

b Mean annual private veterinary expenditures per cow per year (Table 1) added to the total losses due to the endemic diseases and conditions modelled.

## Data Availability

The raw data supporting the conclusions of this article will be made available by the authors, without undue reservation.

## Appendix A

See Table A1 here.
