# Corrigendum to: Estimating the burden of multiple endemic diseases and health conditions using Bayes’ Theorem: A conditional probability model applied to UK dairy cattle [Prev. Vet. Med., vol. 203, June 2022, 105617]

**DOI:** 10.1016/j.prevetmed.2022.105795

**Published:** 2023-01

**Authors:** Philip Rasmussen, Alexandra P.M. Shaw, Violeta Muñoz, Mieghan Bruce, Paul R. Torgerson

**Affiliations:** a Vetsuisse Faculty, University of Zurich, Zurich, Switzerland; b Department of Livestock and One Health, University of Liverpool, Liverpool, United Kingdom; c School of Veterinary Medicine, Centre for Biosecurity and One and Health, Murdoch University, Murdoch, Australia

The authors regret an incorrect citation in Table 7, Row 10. The total economic impact estimate for dystocia has been updated to reflect a 1997 estimate from Dematawena and Berger (1997) that was reported in Kaya et al. (2015) and incorrectly attributed to the 2015 publication. This has resulted in an updated value after adjustment for inflation, and thus an updated sum in Table 7, Row 15. This updated sum is referenced throughout the paper, resulting in minor corrections to several sentences.

The corrections (italicized) are detailed below:

- In Table 7, row 10: Dystocia *31.92*^i^ *Dematawena and Berger* (1997)
- In Table 7, row 15: Total losses *660.26* Calculated
- In Table 7, footnote i: *1997* estimate of 24.24 US$/cow adjusted for inflation at *68.81*% (Inflation Tool, 2021c) and converted to GBP at 0.78 £ /US$ (World Bank, 2021).
- In the Abstract section: Unadjusted aggregation methods suggested losses 14–*63*% greater.
- In Section 3.3. Economic losses: When directly aggregated using disease-specific total loss estimates from the literature (Table 7), annual losses increased by *63*% to £*660*/cow relative to the estimated de-conflated total losses including veterinary expenditures and increased by *43*% relative to the estimated unadjusted total losses including veterinary expenditures.
- In Section 4. Discussion, paragraph 2: When directly aggregated using per-cow economic loss estimates from the literature, the total annual economic burden due to diseases and health conditions endemic to UK dairy cattle was calculated to be £*660* per cow (Table 7).
- In Section 4. Discussion, paragraph 2: At the herd level, this is equivalent to annual losses of £*98,000*.
- In Section 4. Discussion, paragraph 3: Without de-conflation, unadjusted annual per-cow losses due to endemic diseases including private veterinary expenditures were estimated to be £461 (Table 10), equivalent to annual herd-level losses of £68,000, a *30*% reduction from the directly aggregated estimate.
- In Section 4. Discussion, paragraph 3: When de-conflated to account for inter-disease associations, estimated annual per-cow losses including private veterinary expenditures decreased to £404 (Table 10), equivalent to herd-level losses of £60,000, a *39*% reduction.
- In Section 5. Conclusions: Unadjusted productivity gap aggregation suggested losses 14% greater, while direct linear aggregation suggested losses *63*% greater.
- The following reference was added to the reference section: Dematawena, C.M.B. and Berger, P.J. 1997. Effect of dystocia on yield, fertility, and cow losses and an economic evaluation of dystocia scores for Holsteins. J. Dairy Sci., 80 (1997), pp. 754–761, 10.3168/jds.S0022-0302(97)75995-2

The authors would like to apologise for any inconvenience caused.

