# Supplementary material for: Estimating the burden of multiple endemic diseases and health conditions using Bayes’ Theorem: A conditional probability model applied to UK dairy cattle
Source: Prev Vet Med. 2022 Jun;203:105617. doi: 10.1016/j.prevetmed.2022.105617 (PMC9127345; doi:10.1016/j.prevetmed.2022.105617)
Supplement: Supplementary file 1 — Supplementary material. [file mmc1.docx]

**Supplementary File: Estimating the Burden of Multiple Endemic Diseases and Health Conditions Using Bayes’ Theorem: A Conditional Probability Model Applied to UK Dairy Cattle**

**1. Introduction**

Consider a production system with three endemic diseases: Disease 1, Disease 2, and Disease 3. The characteristics of these endemic diseases are described in **Table S1** below.

**Table S1.** Characteristics of endemic diseases in a hypothetical production system.

| Characteristic | Disease 1 | Disease 2 | Disease 3 |
| --- | --- | --- | --- |
| Prevalence  (% of animals) | $P\left( 1 \right)=$10.00 | $P\left( 2 \right)=$15.00 | $P\left( 3 \right)=$ 20.00 |
| Yield impact  (% reduction) | $m_{1}=$2.50 | $m_{2}=$5.00 | $m_{3}=$7.50 |

The statistical associations between disease pairs are described in **Table S2** below.

**Table S2.** Inter-disease odds ratios (ORs) across endemic diseases pairs in a hypothetical production system.

| Disease pair | OR |
| --- | --- |
| Disease 1 : Disease 2 | ${OR}_{1,2}=$2.00 |
| Disease 1 : Disease 3 | ${OR}_{1,3}=$1.00 |
| Disease 2 : Disease 3 | ${OR}_{2,3}=$3.00 |

Also, animals in the production system produce a mean value of 10,000 units/animal per year.

**2. De-conflation Example**

*2.1. Solving for the Value of* $\gamma$ *using* $a$*,* $b$*, and* $c$

The first step in the de-conflation process is to solve for the values of $a$, $b$, and $c$ of the quadratic function of $\gamma$, as described in equations (8), (9), and (10) of the manuscript.

**For Disease 1**, which has a positive statistical association only with Disease 2, the following calculations are required:

From equation (8):

$$a=\left( {OR}_{1,2}-1.00 \right)*P\left( 2 \right)=\left( 2.00-1.00 \right)*0.15=0.15$$

From equation (9):

$$b=-\left[ \left( {OR}_{1,2}-1.00 \right)*\left[ P\left( 1 \right)+P\left( 2 \right) \right]+1 \right]=-\left[ \left( 2.00-1.00 \right)*\left[ 0.10+0.15 \right]+1.00 \right]=-1.25$$

From equation (10):

$$c={OR}_{1,2}*P\left( 1 \right)=2.00*0.10=0.20$$

Lastly, we solve for the roots of the quadratic function of $\gamma$ using the calculated values of $a$, $b$, and $c$, as described in equation (11) of the manuscript.

$$\gamma_{+}=\frac{-b+\sqrt{b^{2}-4*a*c}}{2*a}= -\frac{-\left( -1.25 \right)+\sqrt{\left( -1.25 \right)^{2}-4*\left( 0.15 \right)*\left( 0.20 \right)}}{2*\left( 0.15 \right)}=8.17$$

$$\gamma_{-}=\frac{-b-\sqrt{b^{2}-4*a*c}}{2*a}= -\frac{-\left( -1.25 \right)-\sqrt{\left( -1.25 \right)^{2}-4*\left( 0.15 \right)*\left( 0.20 \right)}}{2*\left( 0.15 \right)}=0.16$$

Since $\gamma=P(i|k)$, as described in equation (5) of the manuscript, only the negative root $\gamma_{-}$ provides a reasonable solution such that $0<P\left( 1 | 2 \right)<1$. Therefore, the value of $P(1|2)$ must be 0.16.

**For Disease 2**, which has positive statistical associations with Disease 1 and Disease 3, the following calculations are required due to its association with Disease 1:

$$a=\left( {OR}_{1,2}-1.00 \right)*P\left( 1 \right)=\left( 2.00-1.00 \right)*0.10=0.10$$

$$b=-\left[ \left( {OR}_{1,2}-1.00 \right)*\left[ P\left( 2 \right)+P\left( 1 \right) \right]+1 \right]=-\left[ \left( 2.00-1.00 \right)*\left[ 0.15+0.10 \right]+1.00 \right]=-1.25$$

$$c={OR}_{1,2}*P\left( 2 \right)=2.00*0.15=0.30$$

$\gamma_{-}=P\left( 2 | 1 \right)=\frac{-\left( -1.25 \right)-\sqrt{\left( -1.25 \right)^{2}-4*\left( 0.10 \right)*\left( 0.30 \right)}}{2*\left( 0.10 \right)}=0.24$

The following calculations are required due to its association with Disease 3:

$$a=\left( {OR}_{2,3}-1.00 \right)*P\left( 3 \right)=\left( 3.00-1.00 \right)*0.20=0.40$$

$$b=-\left[ \left( {OR}_{2,3}-1.00 \right)*\left[ P\left( 2 \right)+P\left( 3 \right) \right]+1 \right]=-\left[ \left( 3.00-1.00 \right)*\left[ 0.15+0.20 \right]+1.00 \right]=-1.70$$

$$c={OR}_{2,3}*P\left( 2 \right)=3.00*0.15=0.45$$

$$\gamma_{-}=P\left( 2 | 3 \right)=\frac{-\left( -1.70 \right)-\sqrt{\left( -1.70 \right)^{2}-4*\left( 0.40 \right)*\left( 0.45 \right)}}{2*\left( 0.40 \right)}=0.28$$

**For Disease 3**, which has a positive statistical association only with Disease 2, the following calculations are required:

$$a=\left( {OR}_{2,3}-1.00 \right)*P\left( 2 \right)=\left( 3.00-1.00 \right)*0.15=0.30$$

$$b=-\left[ \left( {OR}_{2,3}-1.00 \right)*\left[ P\left( 3 \right)+P\left( 2 \right) \right]+1 \right]=-\left[ \left( 3.00-1.00 \right)*\left[ 0.20+0.15 \right]+1.00 \right]=-1.70$$

$$c={OR}_{2,3}*P\left( 3 \right)=3.00*0.20=0.60$$

$$\gamma_{-}=P\left( 3 | 2 \right)=\frac{-\left( -1.70 \right)-\sqrt{\left( -1.70 \right)^{2}-4*\left( 0.30 \right)*\left( 0.60 \right)}}{2*\left( 0.30 \right)}=0.38$$

*2.2. Solving for the Excess Probabilities*

The second step in the de-conflation process is to solve for the values of the remaining conditional probabilities and to use them to calculate the excess probabilities of disease across disease pairs as described in equations (12) through (14) of the manuscript.

**For Disease 1**, using the value of $P(1|2)$ calculated in the previous section, the following calculations are required due to its association with Disease 2:

From equation (12):

$P\left( 2 | 1 \right)=P\left( 1 | 2 \right)*\frac{P\left( 2 \right)}{P\left( 1 \right)}=0.16*\frac{0.15}{0.10}=0.24$

From equation (13):

$P\left( 2 | \neg1 \right)=\frac{P\left( 2 \right)-P(1|2)*P\left( 2 \right)}{1-P\left( 1 \right)}=\frac{0.15-0.16*0.15}{1-0.10}=0.14$

These conditional probabilities are then used to calculate the excess probabilities of disease across animal groups using equation (14):

$${ep}_{2,1}=P(2|1)-P\left( 2 | \neg1 \right)=0.24-0.14=0.11$$

**For Disease 2**, the following calculations are required due to its association with Disease 1:

$$P\left( 1 | 2 \right)=P\left( 2 | 1 \right)*\frac{P\left( 1 \right)}{P\left( 2 \right)}=0.24*\frac{0.10}{0.15}=0.16$$

$P\left( 1 | \neg2 \right)=\frac{P\left( 1 \right)-P(2|1)*P\left( 1 \right)}{1-P\left( 2 \right)}=\frac{0.10-0.24*0.10}{1-0.15}=0.09$

$${ep}_{1,2}=P(1|2)-P\left( 1 | \neg2 \right)=0.16-0.09=0.07$$

The following calculations are required due to its association with Disease 3:

$$P\left( 3 | 2 \right)=P\left( 2 | 3 \right)*\frac{P\left( 3 \right)}{P\left( 2 \right)}=0.28*\frac{0.20}{0.15}=0.38$$

$$P\left( 3 | \neg2 \right)=\frac{P\left( 3 \right)-P(2|3)*P\left( 3 \right)}{1-P\left( 2 \right)}=\frac{0.20-0.28*0.20}{1-0.15}=0.17$$

$${ep}_{3,2}=P(3|2)-P\left( 3 | \neg2 \right)=0.38-0.17=0.21$$

**For Disease 3**, the following calculations are required due to its association with Disease 2:

$$P\left( 2 | 3 \right)=P\left( 3 | 2 \right)*\frac{P\left( 2 \right)}{P\left( 3 \right)}=0.38*\frac{0.15}{0.20}=0.29$$

$P\left( 2 | \neg3 \right)=\frac{P\left( 2 \right)-P(3|2)*P\left( 2 \right)}{1-P\left( 3 \right)}=\frac{0.15-0.38*0.15}{1-0.2}=0.12$

$${ep}_{2,3}=P(2|3)-P\left( 2 | \neg3 \right)=0.29-0.12=0.17$$

*2.3. De-conflating Impact Estimates*

The final step in the de-conflation process is to solve for the de-conflated disease impacts using the excess probabilities calculated in the previous section, as described in equations (15) and (16) of the manuscript.

For **Disease 1**, the de-conflated yield impact (adjusted for its association with Disease 2) is calculated using equation (15) of the manuscript:

$$m_{1,2}=\frac{m_{1}}{1+\frac{{ep}_{2,1}*m_{2}}{m_{1}}}=\frac{2.50}{1+\frac{0.11*5.00}{2.50}}=2.05$$

where, $m_{1}$ and $m_{2}$ are the unadjusted yield impacts presented in **Table S1** for Disease 1 and Disease 2, respectively.

For **Disease 2**, the de-conflated yield impact (adjusted for its associations with Disease 1 and Disease 2) is calculated using equation (16) of the manuscript:

$m_{2,1+3}=\frac{m_{2}}{1+\frac{{ep}_{1,2}*m_{1}+{ep}_{3,2}*m_{3}}{m_{2}}}=\frac{5.00}{1+\frac{0.07*2.50+0.21*7.50}{5.00}}=3.70$

Where, $m_{3}$ is the unadjusted yield impact presented in **Table S1** for Disease 3.

For **Disease 3**, the de-conflated yield impact (adjusted for its association with Disease 2) is calculated using equation (15) of the manuscript:

$$m_{3,2}=\frac{m_{3}}{1+\frac{{ep}_{2,3}*m_{2}}{m_{1}}}=\frac{7.50}{1+\frac{0.17*5.00}{7.50}}=6.74$$

When converted to proportions, the calculated de-conflated yield impacts are 0.02, 0.04, and 0.07 for Disease 1, Disease 2, and Disease 3, respectively.

**3. Productivity Gap Attribution Example**

*3.1. Solving for the Disease-Free Production Value*

Now that the yield impact estimates have been de-conflated and converted to proportions, they can be used to solve for the disease-free value of annual yield given the currently observed mean value of 10,000 units/animal per year.

From equation (21):

$$x_{h}= \frac{\bar{x}}{1-\left[ m_{1,2}*P\left( 1 \right)+m_{2,1+3}*P\left( 2 \right)+m_{3,2}*P\left( 3 \right) \right]}=\frac{10,000.00}{1-\left( 0.02*0.10+0.04*0.15+0.07*0.20 \right)}=10,225$$

The disease-free potential yield is 10,225 units/animal per year.

*3.2. Attributing the Productivity Gap*

Now that the disease-free value of annual yield has been calculated, the gap between that potential yield and the current observed mean can be attributed to the diseases in the model using equation (22) of the manuscript.

For **Disease 1**, the proportion of the gap attributable to its yield impact is calculated:

$g_{1}=\frac{m_{1,2}*P \left( 1 \right)}{m_{1,2}*P \left( 1 \right)+m_{2,1+3}*P\left( 2 \right)+m_{3,2}*P(3)}*\left( x_{h}-\bar{x} \right)=\frac{0.02*0.10}{0.02*0.10+0.04*0.15+0.07*0.20}*\left( 10,225-10,000 \right)=20.45$

For **Disease 2**, the proportion of the gap attributable to its yield impact is calculated:

$g_{2}=\frac{m_{2,1+3}*P\left( 2 \right)}{m_{1,2}*P \left( 1 \right)+m_{2,1+3}*P\left( 2 \right)+m_{3,2}*P(3)}*\left( x_{h}-\bar{x} \right)=\frac{0.04*0.15}{0.02*0.10+0.04*0.15+0.07*0.20}*\left( 10,225-10,000 \right)=61.36$

For **Disease 3**, the proportion of the gap attributable to its yield impact is calculated:

$g_{3}=\frac{m_{3,2}*P(3)}{m_{1,2}*P \left( 1 \right)+m_{2,1+3}*P\left( 2 \right)+m_{3,2}*P(3)}*\left( x_{h}-\bar{x} \right)=\frac{0.07*0.20}{0.02*0.10+0.04*0.15+0.07*0.20}*\left( 10,225-10,000 \right)=143.18$

20, 61, and 143 units/animal are lost annually due to Disease 1, Disease, 2, and Disease 3, respectively, totaling to a productivity gap of approximately 225 units/animal per year.
